# Paraprobiotics in Non-Surgical Periodontal Therapy: Clinical and Microbiological Aspects in a 6-Month Follow-Up Domiciliary Protocol for Oral Hygiene

**DOI:** 10.3390/microorganisms10020337

**Published:** 2022-02-01

**Authors:** Andrea Butera, Simone Gallo, Maurizio Pascadopoli, Carolina Maiorani, Antonella Milone, Mario Alovisi, Andrea Scribante

**Affiliations:** 1 Unit of Dental Hygiene, Section of Dentistry, Department of Clinical, Surgical, Diagnostic and Pediatric Sciences, University of Pavia, 27100 Pavia, Italy; andrea.butera@unipv.it (A.B.); carolina.maiorani01@universitadipavia.it (C.M.); antonella.milone01@universitadipavia.it (A.M.); 2 Unit of Orthodontics and Pediatric Dentistry, Section of Dentistry, Department of Clinical, Surgical, Diagnostic and Pediatric Sciences, University of Pavia, 27100 Pavia, Italy; 3 Department of Surgical Sciences, Dental School, University of Turin, 0121 Turin, Italy; mario.alovisi@unito.it

**Keywords:** dentistry, oral hygiene, periodontitis, scaling and root planing, probiotics, paraprobiotics, tyndallized probiotics, chlorhexidine, periodontology, clinical trial

## Abstract

Periodontal disease represents a progressive destruction of tooth-supporting tissues. Recently, paraprobiotics are regarded as an adjunctive therapy to the non-surgical Scaling-and-Root-Planing (SRP). The aim of this study is to evaluate the efficacy of two new formulations of paraprobiotics, a toothpaste and a mouthwash, respectively, for the domiciliary hygiene. A total of 40 patients were randomly assigned to the following domiciliary treatments: Group 1 (SRP + Curasept Intensive Treatment 0.2% chlorhexidine) (control) and Group 2 (SRP + Biorepair Peribioma toothpaste + Biorepair Peribioma Mousse mouthwash) (trial). At baseline (T_0_) and after 3 and 6 months (T_1_–T_2_), periodontal clinical (Bleeding on Probing, Probing Pocket Depth, Clinical Attachment Loss, Bleeding Score, Sulcus Bleeding Index, Plaque Index, Approximal Plaque Index, Adherent Gingiva, Gingival Recession, and Pathological Sites) and microbiological parameters (Pathological Bacteria, Saprophytic Bacteria, Enlarged Red Complex, Red Complex, Orange Complex, and counts of *Aggregatibacter actinomycetemcomitans*, *Porphyromonas gingivalis*, *Tannerella forsythensys*, *Treponema denticola*, *Prevotella intermedia*, and *Fusobacterium nucleatum*). The use of the experimental products resulted in a significant reduction of most of the clinical indices assessed, which occurred at a major degree with respect to the conventional chlorhexidine considered as control. Additionally, after 6 months of use, the abovementioned products significantly decreased the percentage of pathological bacteria and the counts of those bacteria constituting the “Red Complex”, more related to the periodontal disease. Accordingly, the paraprobiotics-based products tested in this study seem to represent a valid support to SRP with a benefit on both clinical indices and on specific periodontopathogens.

## 1. Introduction

Periodontal disease represents an inflammation of both soft and hard tooth-supporting tissues which is regarded as one of the major causes of tooth loss worldwide [1]. It originates from an untreated gingivitis, a reversible process linked to bacterial plaque accumulation, clinically characterized by an inflammation of the marginal gum with bleeding on probing. In case this process is not reversed, it may lead to periodontitis, presenting irreversible periodontal attachment loss, pockets, recessions, and eventually tooth mobility and even loss [2].

Several factors have been related to periodontal disease, including smoke [3], leukocyte alterations [4], immunosuppression or drugs [5], diabetes [2], and specific genetic polymorphisms associated with inflammatory processes [6]. Anyway, the abovementioned ones are considered predisposing factors, whereas bacterial plaque formation and accumulation are regarded as the main aetiological factors [7].

Periodontal treatment focuses on the removal of the bacterial deposits in order to reverse the inflammatory process. Scaling and Root Planing (SRP) is the non-surgical gold standard procedure which is aimed at removing dental plaque/calculus as well as at smoothing root surfaces, respectively [8]. The most relevant flaw of SRP is the absence of a long-term effect due to an eventual bacterial recolonization after the therapy [9]. Accordingly, adjunctive treatments have been introduced in addition to SRP, e.g., the use of antibiotics, the photodynamic therapy, the administration of antioxidants, natural compounds [10], supplements (e.g., melatonin) [11], and, in recent years, probiotic therapy [12]. Particularly, this last approach is gaining attention in the scientific community because of the absence of the side effects commonly related to antibiotics intake [13]. In accordance with the Food and Agriculture Organization (FAO) and the World Health Organization (WHO), probiotics are “live microorganisms which when administered in adequate amounts confer a health benefit on the host” [14]. Different mechanisms have been proposed to explain probiotics’ actions, such as the competition with periodontopathogens, the production of antimicrobial substances, the enhancement of the mucosal barrier, and an immunomodulant effect [15].

Despite their efficacy, several concerns on the use of probiotics have been raised in recent years. In particular, the safety of live microorganisms should be taken into account, especially when they are administered to vulnerable people, such as the elderly and immunodeficient individuals [16,17]. Based upon these drawbacks, new products based on non-viable probiotics have been proposed, such as paraprobiotics (tyndallized probiotics) and postbiotics. In particular, paraprobiotics are inactivated microbial cells which thus confer a benefit to the consumer without presenting any risk health risk; they are able to regulate both the adaptive and innate immune systems, to exert an antagonistic effect against pathogens, as well as anti-inflammatory, antiproliferative, and antioxidant action [16]. Probiotics and paraprobiotics should not be confused with postbiotics which include any substance released by or produced through the metabolic activity of the microorganism without containing the viable microorganisms itself [18]. As an example, according to a recent study, a postbiotic-based gel, also containing lactoferrin and Aloe Barbadensis Leaf Juice Powder, resulted in an effective tool for the domiciliary treatment of periodontitis [19]. Moreover, immunoodulants effects of paraprobiotics have been assessed in an in vitro study evaluating cellular and inflammatory parameters [20].

The concept of “biotics” should be also discussed taking into account the more general term “metabiotics”, which is used to describe the structural components of probiotic microorganisms and/or their metabolites and/or signalling molecules with a determined chemical structure able to improve host-specific physiological functions, regulator, metabolic, and/or behaviour reactions connected with the activity of host indigenous microbiota [21,22].

On the basis of the previous considerations, the aim of this randomized clinical trial is to analyse the adjuvant efficacy of two paraprobiotic-based products (toothpaste and mouthwash) proposed in addition to SRP for the improvement of periodontal clinical indices and microbiological parameters. Both the products contain tyndallized probiotics of the genus *Lactobacillus* and *Bifidobacterium.*

The first null hypothesis of the study is that there are no significant differences in clinical indices, whereas the second null hypothesis is that no differences occur for either of the microbiological parameters.

## 2. Materials and Methods

### 2.1. Trial Design

This was a parallel, randomized, active controlled, and single-centre trial with a 1:1 allocation ratio, approved by the Unit Internal Review Board (registration number: 2021-0120) and registered on clinicaltrials.gov (NCT04809831).

#### 2.2.1. Participants

Patients addressing for clinical care to the Unit of Dental Hygiene, Section of Dentistry, Department of Clinical, Surgical, Diagnostic and Pediatric Sciences of the University of Pavia (Pavia, Italy) were enrolled for the study after signing the informed consent. The study started in March 2021 and ended in November 2021. Both interventions and outcomes assessment were conducted at the same Unit.

The inclusion criteria were the following: age 18–70 years; presence of periodontal disease (severity: grade II–III; complexity: grade I–II) according to the latest Classification of Periodontal and Peri-Implant Diseases and Conditions (2017) [23]; and patients with no surgical intervention in the latest 12 months. The following exclusion criteria were considered as well: patients with cardiac pacemaker; patients suffering from neurological disorders; patients suffering from psychological disorders; pregnant women; and intake of anti-inflammatory and antibiotic drugs.

#### 2.2.2. Interventions and Outcomes

At the baseline (T0), patients were asked to sign the informed consent to participate to the study. Subsequently, an operator, previously instructed until reaching a low intra-operator variability, conducted a periodontal assessment by means of a probe (UNC probe 15; Hu-Friedy, Chicago, IL, USA) collecting the following indices: Probing Pocket Depth (PPD) (distance from the gingival margin to the bottom of the gingival sulcus or periodontal pocket, evaluated at 6 sites) [24], Clinical Attachment Loss (CAL) (measurement of the position of the gingival margin in relation to the cemento-enamel junction (CEJ) [24], Bleeding on Probing (BoP) (site-specific assessment of gingival bleeding expressed as percentage of total sites) [24], Bleeding Score (BS) (assessment of gingival bleeding with a 0–4 score per each site) [25], Sulcus Bleeding Index (SBI) (assessment of gingival bleeding with a 0–5 score per each site) [26], Approximal Plaque Index (API) (dichotomous assessment of the presence of plaque) [27], Plaque Index (PI) (evaluation of the presence of plaque with a 0–4 score per site) [28], adherent gingiva (AG) (distance between the mucogingival junction and the projection on the external surface of the bottom of the gingival sulcus) [24], gingival recession (GR) (distance from the coronal margin of the gingiva and the cemento-enamel junction) [24], and the percentage of pathological sites (evaluation of the presence of pathological probes) [27]. Then, the 5 sites with the highest PPD values were chosen to subdue microbiological analysis with BPA Lite test (Biomolecular Diagnostic Srl, Firenze, Italy) [24]. Sites were isolated with cotton rolls and gentle drying with compressed air; sterile paper points were inserted in each site until the bottom of the pocket and left for 60 s. Samples were stored and sent to laboratory Real Time PCR-based test. According to the Manufacturer’s protocol, the DNA extraction was conducted by means of QIAsymphony (QIAGEN, Hilden, Germany). Real-time polymerase chain reaction (PCR) with SYBR Green assays were performed using Rotor-Gene Q (QIAGEN) apparatus to quantify periodontopathogens [24,29]. The kit evaluated the following bacteria: *Aggregatibacter actinomycetemcomitans*, *Tannerella forsythensys*, *Porphyromonas gingivalis*, *Treponema denticola*, *Prevotella intermedia*, and *Fusobacterium nucleatum*. Then, a professional supragingival and subgingival oral hygiene was conducted using a piezoelectric instrument (Multipiezo, Mectron S.p.a, Carasco, Italy) and Gracey curettes (Hu-Friedy, Chicago, IL, USA), followed by supragingival and subgingival application of glycine powders (PROPHYflex Perio Powder, KaVo, Biberach, Germany) with specific handpiece (ProphyFlex 4, KaVo, Biberach, Germany) [24]. Finally, patients were randomly allocated to a Control group and a Trial group according to the domiciliary protocol for oral hygiene with medium bristle electric toothbrush: For the Control group, Curasept Intensive Treatment toothpaste (Curasept S.p.A., Saronno, VA, Italy) containing chlorhexidine 0.20% was used twice a day; in the Trial group, instead, Peribioma toothpaste (Coswell S.p.A., Funo di Argelato, BO, Italy) and Peribioma Mousse mouthwash (Coswell S.p.A., Funo di Argelato, BO, Italy) were both used twice a day. In Table 1, the compositions of the products used for the study are shown.

After 3 (T1) and 6 (T2) months, the microbiological assessment and the periodontal indices were re-evaluated, maintaining the domiciliary protocol for oral hygiene. The protocol of the study is shown in Table 2.

#### 2.2.3. Sample Size

Sample size calculation (Alpha = 0.05; Power = 95%) for two independent study groups, and a continuous primary endpoint was calculated.

The following mathematical formula was used for sample size calculation:

(1) $$\mathrm{Sample}\;\mathrm{size}=\frac{Z_{\left(1-\frac{\alpha }{2}\right)}^{2}p\left(1-p\right)}{d^{2}}$$

where $Z_{\left(1-\frac{\alpha }{2}\right)}$ is the standard normal variate corresponding to 1.96 at 5% type 1 error, *p* is the expected proportion in population expressed as decimal and based on previous studies, and, finally, *d* is the confidence level decided by the researcher and expressed as a decimal, too.

Concerning the variable Bleeding on Probing (primary outcome), the expected difference between the means was supposed to be 20% [30], therefore, 20 patients per group were required for the study.

#### 2.2.4. Randomization and Blinding

By means of a permuted block randomization table provided by the data analyst, 40 patients were randomized into Control and Trial groups. An operator enrolled the participants and executed the professional oral procedures. Based on previously prepared, sequentially numbered, opaque, sealed envelopes (SNOSE), an assistant assigned patients to the respective treatment. The order was randomized. The products used were masked so that patients and operator were blinded. The data analyst was blinded.

#### 2.2.5. Statistical Methods

Data were submitted to statistical analysis with R Software (R version 3.1.3, R Development Core Team, R Foundation for Statistical Computing, Wien, Austria). For each group and variable, the following descriptive statistics were calculated: mean, standard deviation, minimum, median, and maximum. PPD and CAL were calculated in millimetres; BOP, API, PI, PS, Pathogen Bacteria, Enlarged Red Complex, Red Complex, and Orange Complex were calculated in percentage of the Total Bacteria Count; BS, SBI, AG, and GR were calculated with the relative score; finally, Total Bacteria Count as well as the presence of each specific microorganism considered were expressed as number of copies per microliter. Kolmogorov–Smirnov test was used to assess data normality. For each variable, inferential comparisons among groups were performed using ANOVA with post hoc Tukey test. Significance was predetermined for *p* < 0.05 for all statistical tests.

## 3. Results

### 3.1. Participant Flow and Baseline Data

**Figure 1:**
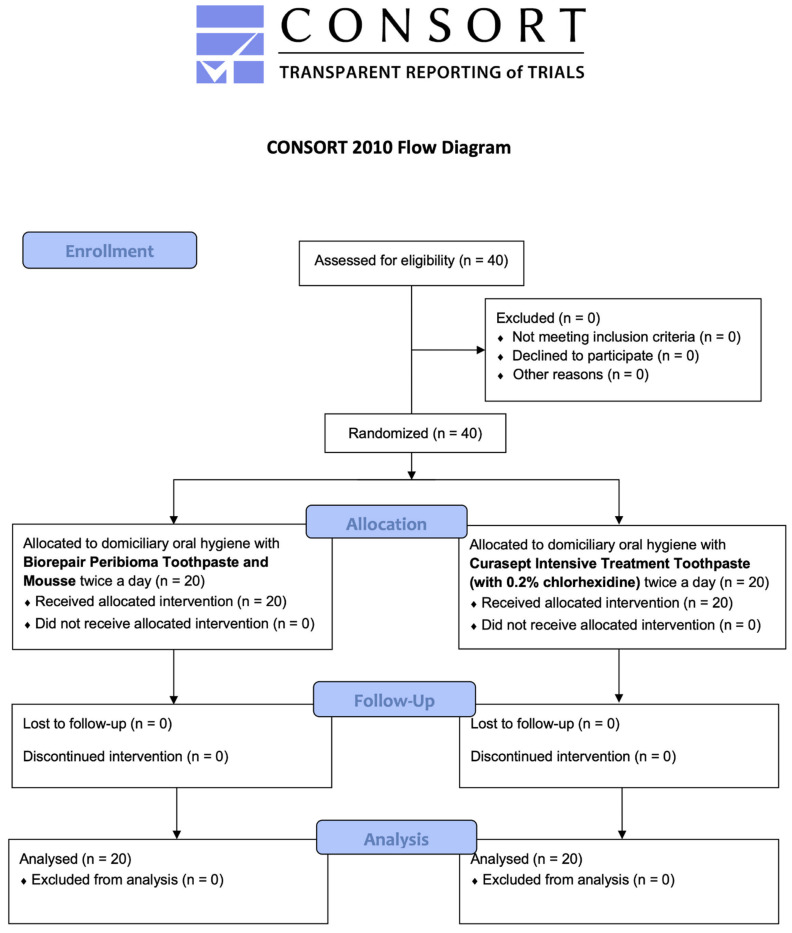
CONSORT flow-chart of the study.

A total of 40 patients responding to the inclusion criteria were asked to participate in the study. They all agreed to participate and received the allocated interventions. No patient was excluded from analysis. The flow chart of the study is shown in Figure 1. At baseline, the sample showed a mean age of 55.2 ± 13.1 years (17 females, mean age 51.6 ± 13.6; 23 males, mean age 57.8 ± 13.7).

Descriptive and inferential statistics are reported in the following sections. Inter- and intra-group comparisons are shown with letter-based comparisons [31].

### 3.2. Periodontal Parameters

**Figure 2:**
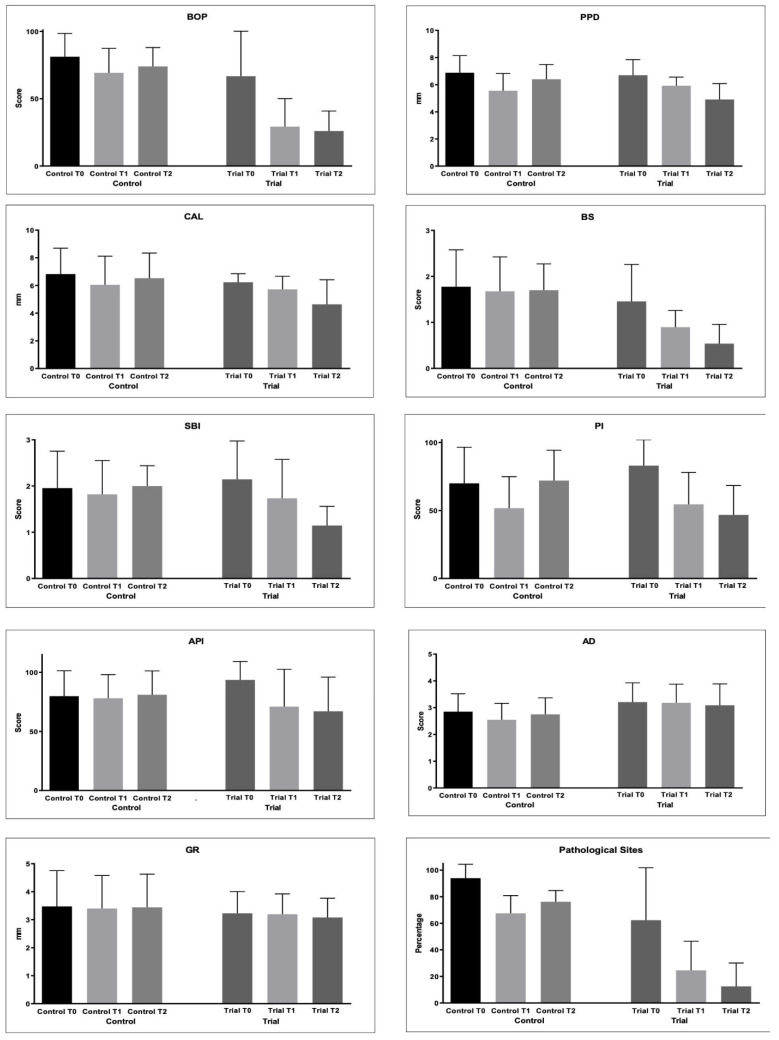
Periodontal parameters collected during the study: Bleeding on Probing (%); Probing Pocket Depth (mm); Clinical Attachment Loss (mm); Bleeding Score (0–4); Sulcus Bleeding Index (0–5); Plaque Index (0–4); Approximal Plaque Index (presence/absence); Adherent Gingiva (mm); Gingival Recession (GR); Pathological Sites (%).

The periodontal indices collected are shown in Table 3 and Figure 2.

BoP decreased in both the groups from baseline to T1 and T2 (*p* < 0.05). However, significantly lower values were found at T1 and T2 in the Trial group with respect to the Control group (*p* < 0.05).

The PPD values significantly decreased in the Trial group from T0 to T1 (*p* < 0.05) and with no significant difference from T1 to T2 (*p* > 0.05). In the Control group, significantly lower values were found at T1 and T2 (*p* < 0.05), but an intergroup comparison at T2 shows significantly lower values in the Trial group after 6 months of treatment (*p* < 0.05).

As regards the CAL values, a significant decrease was found only at T1 in the Control group. In the Trial group, the values significantly decreased until T2 (*p* < 0.05). After 6 months of treatment, significantly lower values were found in the Trial group (*p* < 0.05).

The BS values did not change in the Control group (*p* > 0.05). In the Trial group, the values became significantly lower from T0 and T1 (*p* < 0.05) and from T1 and T2 (*p* < 0.05).

For the SBI values, statistically significantly lower values were found at T1 and T2 if compared to T0 in the Trial group (*p* < 0.05). The same happened for API.

PI significantly decreased at T1 in the Control group (*p* < 0.05), but at T2, a significant increase was observed (*p* < 0.05). In the Trial group, a significant decrease was found at T1 and T2 (*p* < 0.05), with no significant differences from T1 of the Control group (*p* > 0.05).

As regards AG, significantly lower values were found at T1 Control if compared to T0 and T1 of the Trial group (*p* < 0.05). Other comparisons were not significant (*p* > 0.05).

Differences in the GR values among the groups were not significant (*p* > 0.05).

PS significantly decreased in both the groups from T0 to T2 (*p* < 0.05). In the Trial group, significantly lower values were found at T1 and T2 if compared to T1 and T2 of the Control group (*p* < 0.05).

### 3.3. Microbiological Assessment

**Figure 3:**
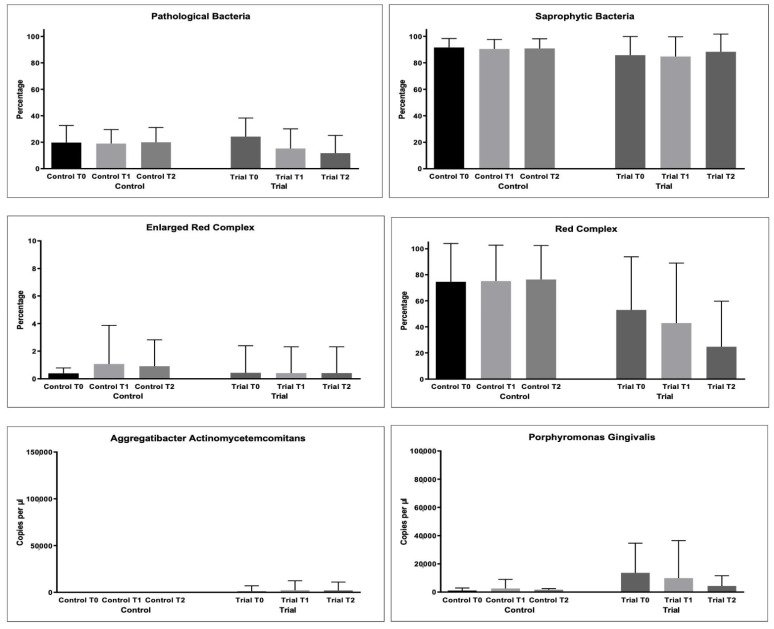

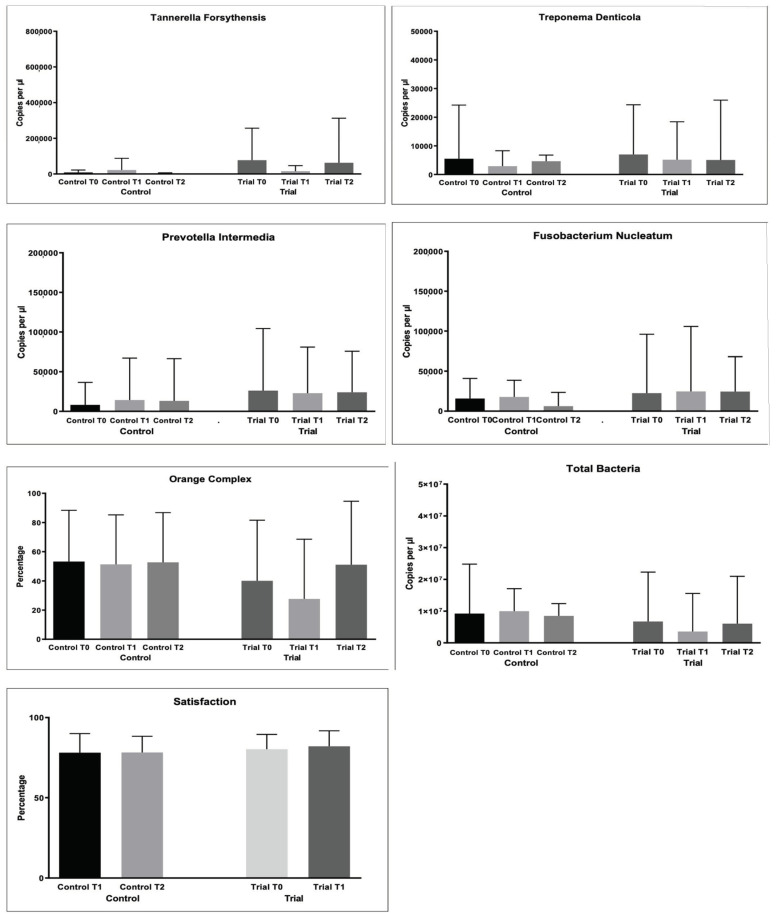
Microbiological variables collected during the study: Pathological Bacteria (%); Saprophytic Bacteria (%); Enlarged Red Complex (%); Red Complex (%); Orange Complex; *Aggregatibacter actinomycetemcomitans* (copies/microliter), *Porphyromonas gingivalis* (copies/microliter), *Tannerella forsythensys* (copies/microliter), *Treponema denticola* (copies/microliter), *Prevotella intermedia* (copies/microliter); *Fusobacterium nucleatum* (copies/microliter).

As regards bacterial samples, multiple comparisons were performed. The percentages of bacterial count, total and per single strain were compared. The results are shown in Figure 3 and Table 4.

For the % of pathological bacteria counts, significantly lower values were found at T2 in the Trial group (*p* < 0.05), as shown in Table 5. The same was found for Red Complex bacteria counts (*p* < 0.05).

For the other microbiological variables, multiple comparisons resulted as non-significant (*p* > 0.05).

## 4. Discussion

The infections of tooth-supporting tissues are responsible for an inflammatory process which can lead susceptible patients to periodontal disease. This is a complex condition where bacteria explain the main pathogenic role, despite several other factors that may also contribute. Interestingly, periodontopathogens do not only influence the local environment but can also have a systemic influence; in particular, according to previous research, they could potentially influence even human behaviour through their capacity to produce and recognize neurochemicals [32,33].

Despite SRP representing the gold standard therapy, the eventual bacterial recolonization represents the major flaw [9]. In light of this, the introduction of adjunctive therapies is strictly required.

Until now, probiotics have been tested in different studies, generally in the form of lozenges with promising clinical results. However, several concerns on the use of living microorganisms have been raised, especially when they are administered to vulnerable people, such as the elderly and immunodeficient individuals; accordingly, paraprobiotics, the heat-inactivated form of probiotics, represent an evolution, assuring benefits to the consumers with no particular health risks [16,17].

The current study arises from previous research of our group where tyndallized probiotics, in form of toothpaste and chewing gum, showed a beneficial action on periodontal clinical indices and periodontal parameters [24,34]. In particular, the aim of the present study is to evaluate another domiciliary hygiene protocol based on a toothpaste and a mouthwash, both containing paraprobiotics of the genus *Lactobacillus* and *Bifidobacterium*.

The first null hypothesis was generally rejected. In fact, the use of the experimental toothpaste and mouthwash resulted in a significant improvement in most of the clinical indices assessed. Conversely, the use of the conventional chlorhexidine, considered as control, caused significant variations only for BoP, PPD, CAL, PI, and PS, generally at a lower rate with respect to the trial group; conversely, BS, SBI, and API did not significantly change at the different timepoints. Accordingly, from a clinical point of view, the combined use of the experimental products tested appear to be an effective domiciliary oral hygiene treatment in additional to non-surgical SRP. As expected, no significant variations resulted in either group for the measures of adherent gingiva (AF) and gingival recession (GR) at any timepoint.

In the literature, studies have mostly tested the effect of probiotics of the genus *Lactobacillus*, both for general and oral diseases. For instance, as regards oral hygiene, a powder containing *Lactobacillus reuteri* (*L. reuteri*) was compared to systemic amoxicillin intake, as adjunctive to SRP, showing a significant improvement in all the clinical indices in both groups [35]. Further studies confirm the benefits of *L. reuteri* with respect to SRP alone, and this is related to a reduction of the inflammatory cytokines and periodontopathogens [36,37,38]. In particular, the findings of the current study confirm those of the previous research of our group where the same toothpaste and an additional paraprobiotic-based chewing gum were tested.

It is important to notice that both the experimental toothpaste and the mouthwash do not only contain bacteria of the genus *Lactobacillus* but also *Bifidobacterium*, despite the specific strains of these bacteria not appearing in the statement of the Manufacturer. Until now, few studies have tested even these microorganisms. A randomized clinical trial demonstrated that lozenges containing *B. lactis* HN019 promote additional clinical, microbiological, and immunological benefits to SRP in case of chronic periodontitis [12]. Accordingly, our results agree with this study, and it may be supposed that *Lactobacillus* and *Bifidobacterium*, both contained in the two products tested here, exert a synergic effect.

The second null hypothesis of the study was partially accepted. Significantly lower values were found at 6 months in the trial group for only the percentage of pathological bacterial counts and for the Red Complex bacteria count. Conversely, multiple comparisons resulted non-significant for the other microbiological variables. Considering the results of our previous research, the paraprobiotics-based treatments did not significantly reduce the percentage of pathogen bacteria, the enlarged red complex and red complex, or the number of copies per microliter of total bacteria, *Aggregatibacter actinomycetemcomitans*, *Tannerella forsythia*, *Porphyromonas gingivalis,* and *Troponema denticola*. Conversely, they managed to significantly decrease the number of bacteria belonging to the orange complex, which is related to periodontal disease, although with a lower grade with respect to the pathogens of the Red Complex. It is interesting to notice that in the current study, conversely, microbiological tests revealed a decrease in the Red Complex but not in the Orange one. It might be supposed that the different oral hygiene protocols considered in the to study could exert a differential effect on the specific microorganisms. In particular, while the association of the paraprobiotics-based toothpaste with the paraprobiotics-based chewing gum seems to have an antimicrobial effect towards the Orange Complex, its association with the mouthwash could be specifically directed towards the Red Complex.

No direct comparisons can be made with data from other authors, due to the lack of extensive research on the efficacy of paraprobiotics-based oral hygiene products on microbiological counts. However, in their randomized clinical trial, Invernici and colleagues [12] compared the effect of *B. lactis* HN019 in form of lozenges, as adjunctive to SRP, with respect to SRP + placebo. The results obtained have shown a significant reduction of the Red Complex after 3 months. Despite the fact that the different methodologies do not allow to directly compare the results obtained, our findings agree with those mentioned above, specifically considering the timing for the reduction of the Red Complex.

Previous studies have evaluated other adjunctive protocols, such as systemic antibiotic intake and photoactivation. For instance, Mocanu and colleagues [39] proposed an investigation on total bacterial load, specific periodontal pathogens, and periodontal clinical parameters in patients with dental fixed prosthesis and different degrees of periodontal tissue loss that followed photoactivation therapy (PDT) adjunctive to Scaling and Root Planing. PDT exerted significant improvements both in clinical and microbiological load after one month, and these results were maintained 6 months after when compared to chlorhexidine rinsing or SRP alone, especially in severe periodontitis cases. As regards antibiotic therapy in non-surgical periodontal therapy, a recent systematic review stated that almost all the clinical trials included yielded superior clinical and microbiological results compared to the placebo group, and the positive effect of the use of antibiotics as an adjunctive to NSPT was assessed regardless of the antimicrobial agents used in the included studies [40]. In particular, sites with PPD > 6 mm may benefit most from the adjunctive use of antibiotics in NSPT.

The novelty of this study is represented by the fact that paraprobiotics have been evaluated as regards their action both on surrogate outcomes (i.e., microbiological counts) as well as on clinical ones. The major limitation of the current study, other than the relatively small sample size, is that no evaluations of a direct colonization of periodontium by beneficial paraproprobiotic bacteria have been carried out. Conversely, only the decrease in periodontopathogens has been measured. Moreover, an eventual low compliance or incorrect domiciliary use of the products by the patients could have altered the results. Finally, the 6-month follow up could be considered relatively short to perform evaluations on the stability of the clinical and microbiological improvements. Particularly, the risk of a long-term bacterial recolonization still represents the major concern of periodontal therapy. On the basis of these considerations, future studies should be conducted with a longer follow up in order to evaluate the efficacy of paraprobiotics in preventing the bacterial recolonization of the periodontal pockets treated. Finally, it could be interesting to compare different protocols (i.e., toothpaste + mouthwash vs. toothpaste + chewing gum) in order to fully understand whether the experimental mouthwash and the chewing gum effectively exert a differential antimicrobial action on those complexes traditionally related to periodontitis. As regards this last point, however, it is important to consider that periodontitis should not be regarded as the simple result of an infection of tooth-supporting tissues, since it also derives from an altered immunological status of the sites affected [41]. Therefore, considering that the substances for non-surgical periodontal treatment should exert not only an inflammatory effect but also an immunomodulant one, future in vitro and clinical studies are required to evaluate the effect of the toothpaste and the mouthwash even on these parameters. Along these lines, promising results have been obtained, encouraging the use of paraprobiotics for their immunomodulant effects [20]. Comparisons with other protocols, such as photoactivation therapy or antibiotic intake, are also necessary. Additionally, the use of probiotics in combination with low-invasive procedures such as ozone [42] and photobiomodulation [43] could add more interesting results to the topic.

## 5. Conclusions

Paraprobiotics represent an innovative adjunctive therapy for the treatment of periodontal disease. Following the clinical procedure of Scaling and Root Planing (SRP), the combination of the experimental toothpaste and mouthwash tested represent an effective protocol for the domiciliary maintenance of oral health. Paraprobiotics-based agents could be particularly relevant thanks to the absence of both contraindications and shortcomings generally related to antibiotics.

## Figures and Tables

**Table 1:** Materials used for the study.

Product	Manufacturer	Composition
Biorepair Peribioma toothpaste	Coswell S.p.A., Funo di Argelato, BO, Italy	Aqua, Sorbitol, Zinc Hydroxyapatite *, Glycerin, Hydrated Silica, Silica, Cocamidopropyl Betaine, Cellulose Gum, Aroma, Sodium Hyaluronate, Hamamelis Virginiana Leaf Extract, Spirulina Platensis Extract, Calendula Officinalis Flower Extract, Bifidobacterium *, Lactobacillus *, Zinc PCA, Benzyl Alcohol, Sodium Benzoate, Phenoxyethanol, Sodium Saccharin, Maltodextrin, Citric Acid, Potassium Sorbate, Sodium Myristoyl Sarcosinate, Sodium Methyl Cocoyl Taurate, Limonene, Eugenol, CI 77891, CI 16255.
Biorepair Peribioma Mousse mouthwash	Coswell S.p.A., Funo di Argelato, BO, Italy	Aqua, Sorbitol, Xylitol, Zinc Hydroxyapatite *, Aroma, Pistacia Lentiscus (Mastic) Gum Oil, Lactobacillus *, Bifidobacterium*,Sodium Hyaluronate, Ascorbic Acid, Hamamelis Virginiana Leaf Extract, Spirulina Platensis Extract, Calendula Officinalis Flower Extract, Tocopheryl Acetate, Retinyl Palmitate, Eucalyptus Globulus Leaf Oil, PEG-40 Hydrogenated Castor Oil, Phenoxyethanol, Sodium Benzoate, Cocamidopropyl Betaine, Glycerin, Maltodextrin, Sodium Saccharin, Helianthus Annuus Seed Oil, Potassium Sorbate, BHT, Limonene, CI 16255.
Curasept Intensive Treatment (0.2% chlorhexidine) toothpaste	Curasept S.p.A., Saronno, VA, Italy	Purified Water, Sorbitol, Hydrated Silica, PEG-32, Cocamidopropyl Betaine, Xylitol, Cellulose Gum, Aroma, Sodium Hyaluronate, Ascorbic Acid, Chlorhexidine, Digluconate, Sodium Metabisulfite, Sodium Citrate, Titanium Dioxide (C.I. 77891), Sodium Benzoate, Sodium Saccharin, Citric Acid, C.I. 17200, C.I. 42090.

* Zinc Hydroxyapatite, Bifidobacterium, and Lactobacillus constitute the complex microRepair^®^BIOMA.

**Table 2:** Protocol adopted for the study.

Appointment	Procedures
	Signature of the informed consent for the study
	Collection of periodontal indices and bacterial assessment
	Professional supragingival and subgingival oral hygiene
Baseline (T0)	Patients’ randomization for domiciliary procedures of oral hygiene with medium bristle electric toothbrush:
	*Control Group*: use of Curasept Intensive Treatment Toothpaste (with 0.2% chlorhexidine) twice a day;
	*Trial Group*: use of Biorepair Peribioma Toothpaste and Mousse twice a day.
After 3 months (T3) After 6 months (T6)	Collection of periodontal indices and bacterial assessment Patients continue with domiciliary protocol of oral hygiene in both Control and Trial groups.

**Table 3:** Descriptive statistics of periodontal clinical parameters.

Group	Time	BOP	PPD	CAL	BS	SBI	API	PI	AG	GR	PS
		Mean	Mean	Mean	Mean	Mean	Mean	Mean	Mean	Mean	Mean
		(SD)	(SD)	(SD)	(SD)	(SD)	(SD)	(SD)	(SD)	(SD)	(SD)
**Control**	T_0_	81.25 (17.23) ^a^	6.88 (1.26) ^a,d^	6.83 (1.87) ^a^	1.78 (0.80) ^a^	1.96 (0.80) ^a^	79.75 (21.61) ^a^	70.00 (26.56) ^a,b,c^	2.85 (0.67) ^a,b^	3.48 (1.28) ^a^	94.00 (10.46) ^a^
	T_1_	69.25 (18.16) ^b^	5.55 (1.27) ^b,e^	6.05 (2.07) ^b,c^	1.68 (0.75) ^a^	1.82 (0.73) ^a^	78.00 (20.03) ^a^	51.75 (23.13) ^b^	2.55 (0.61) ^b^	3.40 (1.18) ^a^	67.50 (13.33) ^b^
	T_2_	74.00 (14.01) ^b^	6.40 (1.08) ^c,f^	6.53 (1.82) ^a^	1.70 (0.57) ^a^	2.00 (0.44) ^a^	81.00 (20.17) ^a^	72.00 (22.33) ^a,c^	2.75 (0.62) ^a,b^	3.44 (1.19) ^a^	76.25 (8.41) ^c^
**Trial**	T_0_	66.75 (33.41) ^a,b^	6.69 (1.15) ^b,c,d^	6.23 (0.62) ^a,b^	1.46 (0.80) ^a^	2.15 (0.83) ^a^	93.50 (15.65) ^a^	83.00 (18.95) ^c^	3.21 (0.72) ^a^	3.23 (0.78) ^a^	62.35 (39.55) ^b,c^
	T_1_	29.25 (20.82) ^c^	5.92 (0.64) ^a,e,f^	5.73 (0.94) ^a,b^	0.90 (0.36) ^b^	1.74 (0.84) ^a,b^	71.00 (31.48) ^a,b^	54.50 (23.50) ^a,b,d^	3.18 (0.70) ^a^	3.20 (0.73) ^a^	24.55 (21.98) ^d^
	T_2_	26.00 (14.85) ^c^	4.91 (1.18) ^e^	4.64 (1.78) ^c^	0.54 (0.42) ^c^	1.15 (0.41) ^b^	67.00 (28.95) ^b^	46.75 (21.66) ^b,d^	3.09 (0.80) ^a,b^	3.08 (0.69) ^a^	12.46 (17.62) ^d^

For each variable tested, groups with different letters (a, b, c, d, e and f) show significantly different means between them.

**Table 4:** Descriptive statistics of microbiological parameters (1).

Group	Time	Total Bacteria Count	AAE	PG	TF	TD	PI	FN
		Mean	Mean	Mean	Mean	Mean	Mean	Mean
		(SD)	(SD)	(SD)	(SD)	(SD)	(SD)	(SD)
**Control**	T_0_	9,225,901.65 (15,558,308.5) ^a^	0.83 (2.20) ^a^	1053.05 (1842.44) ^a^	8416.22 (13584.71) ^a^	5505.00 (18730.81) ^a^	8295.00 (28278.05) ^a^	15,792.48 (25232.49) ^a^
	T_1_	9,986,050.23 (7,074,899.18) ^a^	0.64 (2.61) ^a^	2567.22 (6425.14) ^a^	21,956.85 (65,796.72) ^a^	2959.11 (5366.56) ^a^	14,227.38 (52,965.73) ^a^	17,811.83 (20,803.62) ^a^
	T_2_	8,502,000.00 (3,858,474.75) ^a^	1.05 (2.14) ^a^	1695.80 (794.89) ^a^	4099.00 (1993.82) ^a^	4651.00 (2158.87) ^a^	13,304.81 (53,130.25) ^a^	6275.86 (17,221.46) ^a^
**Trial**	T_0_	6,732,956.50 (15,565,648.5) ^a^	1315.00 (5669.24) ^a^	13,634.51 (21,032.74) ^a^	77,338.46 (179,432.46) ^a^	7023.45 (17,331.05) ^a^	26,163.01 (78,205.03) ^a^	22,547.43 (73,550.18) ^a^
	T_1_	3,577,611.70 (12,004,436.6) ^a^	2300.00 (10,074.20) ^a^	9933.55 (26,547.19) ^a^	14,984.63 (31,781.70) ^a^	5189.00 (13,216.46) ^a^	22,916.50 (58,000.75) ^a^	24,758.25 (81,217.50) ^a^
	T_2_	6,038,339.10 (14,922,620.2) ^a^	2045.00 (8933.82) ^a^	4362.55 (7230.12) ^a^	62,514.97 (250,286.56) ^a^	5095.50 (20,882.84) ^a^	24,086.56 (51,657.98) ^a^	24,483.45 (43,599.34) ^a^

For each variable tested, groups with the same letter (a) show no significantly different means between them.

**Table 5:** Descriptive statistics of microbiological parameters (2).

Group	Time	Pathogen Bacteria	Enlarged Red Complex	Red Complex	Orange Complex
		Mean	Mean	Mean	Mean
		(SD)	(SD)	(SD)	(SD)
**Control**	T_0_	19.71 (12.93) ^a^	0.4	74.58	53.27
			(0.39) ^a^	(29.41) ^a^	(35.14) ^a^
	T_1_	18.92 (10.64) ^a^	1.08	75.19	51.38
			(2.79) ^a^	(27.52) ^a^	(33.83) ^a^
	T_2_	19.90 (11.20) ^a^	0.91	76.38	52.69
			(1.91) ^a^	(26.10) ^a^	(34.08) ^a^
**Trial**	T_0_	24.20 (14.09) ^a^	0.44	52.95	40.09
			(1.95) ^a^	(40.85) ^a^	(41.49) ^a^
	T_1_	15.24 (14.87) ^a,b^	0.42	42.86	27.66
			(1.89) ^a^	(46.10) ^a,b^	(40.87) ^a^
	T_2_	11.70 (13.40) ^b^	0.42	24.69	51.09
			(1.89) ^a^	(35.02) ^b^	(43.52) ^a^

For each variable tested, groups with different letters (a and b) show significantly different means between them.

## Data Availability

All data are available upon request to corresponding authors.
